# Conference Reports: CIMCON’90 CONFERENCE—COMPUTER INTEGRATED MANUFACTURING (CIM) ARCHITECTURE CONFERENCE (CON) Gaithersburg, MD May 22–24, 1990

**DOI:** 10.6028/jres.095.048

**Published:** 1990

**Authors:** Albert Jones

**Affiliations:** Center for Manufacturing Engineering, National Institute of Standards and Technology, Gaithersburg, MD 20899

The National Institute of Standards and Technology Center for Manufacturing Engineering together with the Navy Manufacturing Technology Program and the Air Force WRDC/MTI program jointly sponsored the first conference to address the problems associated with designing and implementing a system architecture for Computer Integrated Manufacturing (CIM). More than 125 people attended from the United States, Canada, Denmark, England, France, Italy, Japan, the Netherlands, Switzerland, the Soviet Union, and West Germany. The conference was held in conjunction with a meeting of the international standards committee ISO TC 184/SC 5/WG 1. This committee is looking into the possibility of developing a standard framework for CIM.

## 1. About the Conference

Two considerations motivated this conference. First, the initial implementation phases of early CIM programs had been completed and a great deal had been learned from those experiences. The conference provided a forum for people to describe their original architectures and implementation plans, to discuss what did and did not work, and to report on changes they might be considering in those architectures and plans.

Second, the ISO TC 184/SC 5/WG 1 committee had just been assigned a new work item to develop a CIM architecture to aid in identifying standards needed to enable integration of all systems within the CIM enterprise. The conference gave those responsible for developing that architecture the opportunity to discuss this issue with experts from around the world and to learn about the problems involved in integrating new and existing manufacturing systems.

The conference had three major goals: to propose some architectures, to examine some implementation problems, and to discuss some design tools.

## 2. Opening the Conference

Albert Jones, Conference chairman and Deputy Program Director of the Automated Manufacturing Research Facility (AMRF) welcomed the conference participants. John Lyons, Director of NIST, gave the opening address. In his address, Dr. Lyons pointed out that the conference provided an opportunity for speakers to discuss research and implementation issues related to architectures for computer integrated manufacturing. He emphasized that NIST has been involved in these areas for a number of years through the AMRF and other programs. He then discussed the new mission and direction of NIST and some of the new projects underway at NIST. Dr. Lyons concluded by stressing the importance of completing the research, development, and technology transfer chain and expressing his hope that the conference would strengthen that chain.

## 3. Architectures

Albert Jones and Edward Barkmeyer gave the opening paper at the conference. They described the control hierarchy, the distributed data management system, and the communications system developed in the AMRF program at NIST. Sanjay Joshi from Penn State then described how one could use control grammars to implement the control hierarchy described by Jones. He also indicated how one could decompose process plans to match the tasks assigned to the levels in that control hierarchy. Robert Boykin from CAM-I gave an overview of CAM-I’s work in CIM architecture development and CIM standards in general. Clyde Van Haren, James River Corporation, presented the Reference Model developed by the International Purdue Workshop. This model includes a hierarchy for shop floor control and data flow diagrams to capture information flows. This strategy was also used to develop the RAMP architecture described by David Jung. Paul Fehrenbach of GEC Marconi Research Centre, UK, described his DtoP (Design to Product) architecture developed as part of an ESPRIT project. Allan Anderson of Honeywell gave an overview of the architectural approach being developed at Honeywell. Michel Böhms of TNO Netherlands gave some criteria for comparing various architectures.

Several European authors described a different approach based on the “matrix concept.” The oldest and most famous of these, called CIM-OSA, was described by Richard Panse from IBM Germany. The CIM-OSA framework was developed by the ESPRIT AMICE Consortium. The framework is a three dimensional matrix. One dimension contains three modeling levels: requirements definition, design specification, and implementation description. The second dimension contains three levels of genericity: Generic, Partial, and Particular. The last dimension contains four views: Function, Information, Resource, and Organization. David Shorter, SD Scicon UK, compared the CIM-OSA model with the model developed in the ISO standards committee ISO TC 184/SC 5/WG 1. Bruno Vallespir, GRAI Laboratory France, also described a 36 cell matrix but with different definitions for the dimensions. Wolfram Süssenguth, Fraunhofer Institut Berlin, presented a framework that combined the CIM-OSA matrix with the WG 1 Reference Model.

Shop floor control was addressed by several authors. Two approaches were advocated: heterarchical and hierarchical. In the former, decisions are arrived at by a committee of cooperating agents. James Ting of Michigan University and Matt Johnson of DEC Italy discussed the advantages of this approach at length. In hierarchical control, entities are arranged in a tree structure with each entity having a single supervisor. Frank Biemans and Roli Wendorf of North American Philips discussed the advantages of this approach.

Both Mukasa Ssemakula from the University of Maryland and Wayne Davis of the University of Illinois described architectures for integrating higher level functions such as CAD, CAPP, MRP-II, and production planning with shop floor architectures.

## 4. Design Tools

Robert Young of NC State University described the IDEF_0_ and IDEF_1_ modeling tools and how these tools were used to upgrade existing manufacturing plants in Denmark. Bruno Vallespir reported on his efforts to integrate the IDEF tools with the French-developed GRAI tools to fill in the cells of his architecture. John Sauter of ITI described XSpec, a graphical tool which can represent both physical and control components and the messages that flow between them. Hugh Sparks from MTS Systems Corporation has integrated a graphical dataflow language and object-oriented programming techniques into a design tool called HOSE which is portable to a variety of hardware platforms. Larry Zeidner of Boston University presented the Server Network Generator which provides a distributed software development environment which allows the designer to separate the complexity of individual components from the complexity of their interconnection.

## 5. Implementation Issues

Richard Weston of Loughborough University, UK, described the AUTOMAIL system as an alternative to the “hard-wired” approach being used in industry today. He gave two examples of how AUTOMAIL was used to build an integrated printed circuit board cell and manufacturing cell at a major UK computer systems company. Dirk Beeckman of Gap Gemini Sesa, Belgium, provided some results of a recent attempt to use the CIM-OSA concepts to model the FMS in the FIAT Mirafiori plant. He concentrated on the Function and Information views for the Design Specification and Requirements Definition cells in the CIM-OSA matrix. David Jung of Battelle reviewed the lesson learned from attempts to adapt the RAMP architecture to the small mechanical parts cell at the Cherry Point Naval Aviation Depot. Major Knute Hankins, U.S. Army Watervliet Arsenal, discussed the problems involved in integrating off-the-shelf software products and verifying the accuracy of the data that was used to run those packages. John Ettlie of Michigan University used several case studies to give an overview of some of the problems facing industry as they try to automate and integrate.

## 6. For More Information

Proceedings of CIMCON’90 (NIST Special Publication 785) is available from the Superintendent of Documents, U.S. Government Printing Office, Washington, DC 20402. Order by stock no. 003-003-03010-4 (phone 202/738-3238).

